# Detection, Distribution and Health Risk Assessment of Toxic Heavy Metals/Metalloids, Arsenic, Cadmium, and Lead in Goat Carcasses Processed for Human Consumption in South-Eastern Nigeria

**DOI:** 10.3390/foods10040798

**Published:** 2021-04-08

**Authors:** Emmanuel O. Njoga, Ekene V. Ezenduka, Chiazor G. Ogbodo, Chukwuka U. Ogbonna, Ishmael F. Jaja, Anthony C. Ofomatah, Charles Odilichukwu R. Okpala

**Affiliations:** 1 Department of Veterinary Public Health and Preventive Medicine, Faculty of Veterinary Medicine, University of Nigeria, Nsukka 410001, Nigeria; ekene.ezenduka@unn.edu.ng (E.V.E.); kiaradollz21@gmail.com (C.G.O.); 2 Department of Biochemistry, Federal University of Agriculture Abeokuta, Ogun State 110124, Nigeria; ogbonnacu@funaab.edu.ng; 3 Department of Livestock and Pasture Science, University of Fort Hare, Alice 5700, South Africa; ijaja@ufh.ac.za; 4 National Centre for Energy Research and Development, University of Nigeria, Nsukka 410001, Nigeria; ofomatony@yahoo.co.uk; 5 Department of Functional Food Products Development, Faculty of Biotechnology and Food Science, Wrocław University of Environmental and Life Sciences, 51-630 Wrocław, Poland

**Keywords:** arsenic (As), cadmium (Cd), lead (Pb), consumer food safety, goat meat, health risk, toxic heavy metals

## Abstract

Notwithstanding the increased toxic heavy metals/metalloids (THMs) accumulation in (edible) organs owed to goat′s feeding habit and anthropogenic activities, the chevon remains increasingly relished as a special delicacy in Nigeria. Specific to the South-Eastern region, however, there is paucity of relevant data regarding the prevalence of THMs in goat carcasses processed for human consumption. This work was, therefore, aimed to investigate the detection, distribution and health risk assessment of THMs in goat carcass processed for human consumption in South-Eastern Nigeria. To achieve this, a total of 450 meat samples (kidney, liver and muscle) were evaluated from 150 randomly selected goat carcasses processed in two major slaughterhouses in Enugu State. The detection, distribution, as well as health risk assessment parameters followed standard procedures. Results revealed that at least one THM was detected in 56% of the carcasses. Mean concentrations of arsenic (As) were 0.53 ± 0.10 mg/kg, 0.57 ± 0.09 mg/kg and 0.45 ± 0.08 mg/kg, lead (Pb) were 0.48 ± 0.38 mg/kg, 0.45 ± 0.24 mg/kg and 0.82 ± 0.39 mg/kg, cadmium (Cd) was 0.06 ± 0.32 mg/kg, 0.02 ± 0.00 mg/kg, and 0.02 ± 0.00 mg/kg for kidney, liver and muscle tissues, respectively. The estimated daily intakes (EDI) for all THMs were above the recommended safe limits. The target hazard quotient (THQ) and hazard index (HI) computed for all As, Cd and Pb fell below unity in all the studied organs, which indicated no non-carcinogenic risks. Curtailing the anthropogenic activities that aid the THM-contamination in goat production/processing lines is recommended. Screening for THM-contamination in Nigerian slaughterhouses is imperative, so as to ascertain the toxicological safety of meats intended for human consumption.

## 1. Introduction

Heavy metals/metalloids (HMs) entail chemical elements with a density greater than 5 g/cm^3^ or specific gravity of at least five times greater than that of water [1]. Required in minute quantities for homeostasis and optimal body function, excess or prolonged intake of such micronutrients like Zn, Fe, Cu, Mn, Co, Mo, and Cr may result in adverse health conditions [2]. Toxic heavy metals/metalloids (THMs) refer to relatively dense metals/metalloids not actually beneficial in both humans and livestock, which in small amounts can lead to carcinogenicity, organ toxicity and other deleterious health effects [1,3,4,5,6,7,8,9]. Examples like arsenic (As), cadmium (Cd), lead (Pb) and mercury (Hg) are among the top ten THMs of major public health concern by the World Health Organization (WHO) of the United Nations [10]. The toxicity of THMs has always been dependent on the dose, route of exposure, age and nutritional statuses of the exposed individuals or animals [11,12]. Multiple industrial, domestic, agricultural, medical and technological uses of THMs may result in heavy environment pollution with the toxic metals and the possibilities of food-producing animals (FPAs) and humans getting exposed [13]. HM-contaminated pasturelands and water outlets, and indiscriminate use of acaricides, are among THMs entry pathways in edible tissues of FPAs [14,15] for onward transmission to humans via the food chain. Notwithstanding that THMs may naturally occur in minute quantities in the environment, certain anthropogenic activities can exacerbate the pollution of pastureland or water outlets used for food-animal production, especially with toxic metals [16,17].

**Figure 1:**
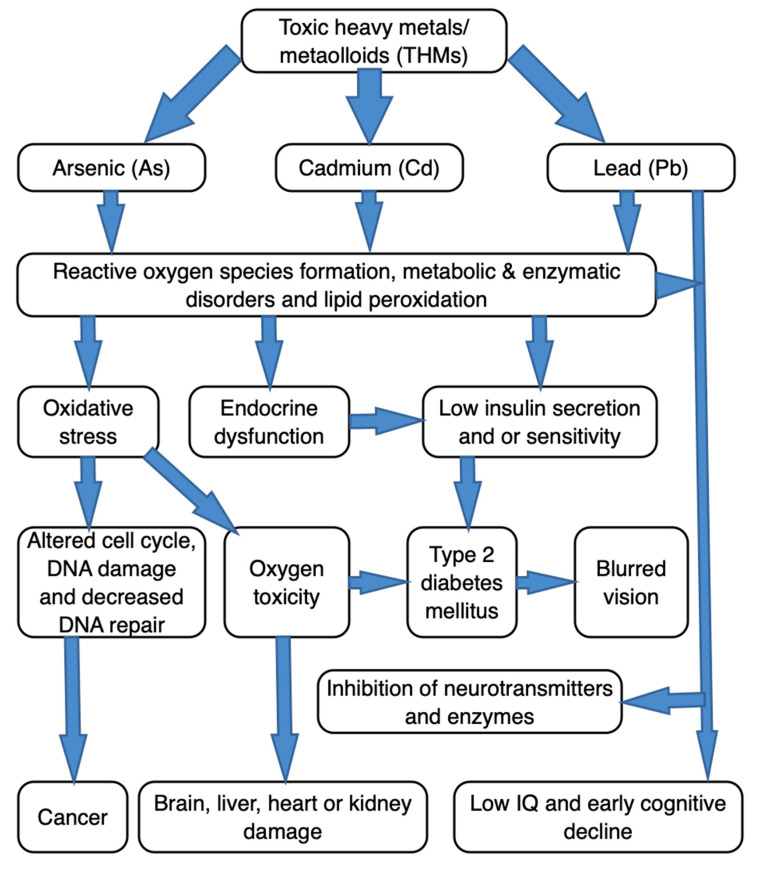
A schematic diagram depicting the complexities associated with toxic heavy metals (also ‘metalloids’), arsenic, cadmium and lead, in the pathogenesis and progression of certain disease conditions of public health importance.

In Nigeria, the environmental contamination with THMs could increase given anthropogenic activities like: (a) the indiscriminate (agricultural) use of agrochemical; (b) unregulated artisanal mining; (c) crude oil spillage due to oil pipeline vandalism; (d) natural gas flaring; (e) excessive use of explosives (bombs and other improvised explosive devices) associated with crisis like insurgency and or terrorism; and (d) improper disposal of industrial effluents. Environmental contamination with THMs undoubtedly affects pastures, fodder plants, or drinking water sources [16] used for livestock production where the soil serves as platform for either phytoremediation or active uptake [18,19]. A schematic diagram depicting the complexities associated with As, Cd and Pb as THMs, from pathogenesis to progression of certain disease conditions, is shown in Figure 1. Indeed, these THMs can bring about a number of adverse health effects, and in diverse ways. The onset and type of clinical manifestations associated with THMs-toxicity depend on the type of metal involved and the affected organ. Unstable chemically, THMs are able to bind with the biomolecules (proteins, enzymes, phospholipids, hormones and other tissue constituents) to form other chemical complexes [20,21]. Such (THMs-biomolecule) complexes may result in either irreversible or detrimental alterations in its biochemistry, transport, neurotransmission or signaling systems particularly within the affected host [1,20,21,22]. Further, the THMs can interact with the cellular components, for instance, DNA and nuclear proteins, and bring about conformational changes, which could appear in a form of carcinogenesis or detrimental cell cycle modulation [23,24,25]. Additionally, an organ failure or malfunction could arise. This is because THMs in both bound and unbound forms could accumulate in vital organs, like kidney, brain, and liver [13,26]. Whether in the environment, or in the animal/human tissue, the THMs remain among crucial chemical pollutants, given its bio-accumulation/magnification potentials occasioned by diminutive (tissue) biodegradability [27]. Excessive amounts of THMs in edible tissues of food animals would either directly indicate the degree of meat′s toxicological safety, or indirectly indicate the environmental contamination or pollution of toxic metals. Given their voracious appetite and low-grazing feeding habit, goats (*Capra hircus*) are particularly prone to ingestion of THMs during grazing [28]. The peculiar feeding habits of goat may facilitate geophagia, which increases its propensity to ingest THMs deposited on the soil surface, especially if grazing a contaminated pastureland.

Given the food safety concerns associated with accumulated THMs, international food safety regulatory authorities like those under the European Commission [29] have set maximum permissible limits (MPL) for the metals in edible animal products. It is illegal for THMs to occur in edible tissues above the stipulated MPL given the substantial health risks and adverse health effects its exposures pose to human. Typical example includes “itia-itia” (it hurts, it hurts) disease first reported in Toyama Prefecture, Japan, around 1912, owed to the mass Cd poisoning, characterized by osteomalaecia with excruciating joint and spine pains and kidney dysfunction. Additionally, the ingestion of THMs in food or water can result in organ dysfunction and carcinogenicity [1,30,31,32]. With about 74 million goats as of 2016, Nigeria is the highest producer of goat in Africa, the third largest producer globally, and the second largest chevon consumer (in Africa) [33,34]. Meat offals like kidneys and liver are edible and increasingly preferred across several communities in Africa [35]. Since THMs bio-accumulate in these organs (that is, kidneys and liver) [17,36], an active surveillance is warranted to safeguard public health. Notwithstanding, the increased THMs accumulation in (edible) organs owed to goat′s feeding habit and anthropogenic activities, the chevon remains increasingly relished as a special delicacy in Nigeria. Thus, the indiscriminate anthropogenic activities that result in environmental contamination, and subsequent accumulation of THMs [37,38] makes the detection and quantification of toxic metals in goat carcasses processed for human consumption in Nigeria imperative. The work by Okoye and Ugwu [39], to our best knowledge, appears to be the only recent report on toxic metal contamination of goat muscles and edible offals, which was specific to the South-Eastern region of Nigeria, and this was conducted over a decade ago.

Despite that contamination of natural water bodies/pasturelands used for goat production continues to persist, the demand as well as consumption specific to goat carcass in various region of Nigeria continues to increase [40]. Previous studies largely in Northern region of Nigeria, on THM contamination in FPAs like cattle, camel, goat and sheep slaughtered for human consumption have been reported [32,41,42,43]. Specific to the South-Eastern region however, there is paucity of relevant data regarding the prevalence of THMs in goat carcasses processed for human consumption. This paucity of relevant data may be contributing to the impediment on establishment as well as implementation of food safety regulations regarding (THMs) contamination in goat carcasses, especially with respect to chevon. Besides, in most Nigerian slaughterhouses, unfortunately, the screening for THMs in meats appears not standardly conducted during the routine meat inspection processes, and this needs to change. To supplement existing information, therefore, this work was aimed to investigate the detection, distribution and health risk assessment of THMs in goat carcass processed for human consumption in Enugu State, which is in South-Eastern Nigeria. The target is to see if we can acquire a better understanding about the current status of goat carcass contamination with THMs, as well as some additional insights about its toxicological safety, specific to the South-Eastern Nigeria.

## 2. Materials and Methods

### 2.1. Schematic Overview of Experimental Program

**Figure 2:**
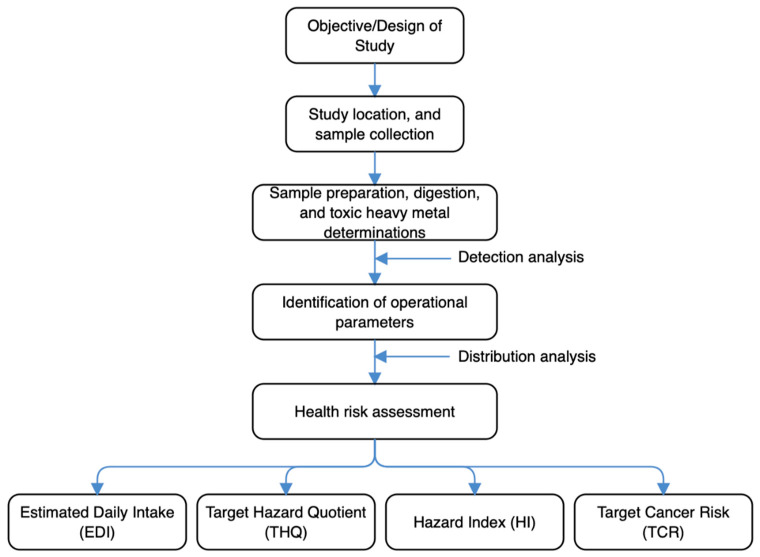
The schematic overview of the experimental program, from design of study, study location and sample collection, its preparation, digestion, and toxic heavy metals/metalloids (THMs) determinations, identification of operational parameters, to the health risk assessment.

The schematic overview of the experimental program, from design of study, study location and sample collection, its preparation, digestion, and THM determinations, identification of operational parameters, to the health risk assessment, are depicted in Figure 2. For emphasis, this current work has been designed to understand the extent THMs would be detected in goat carcasses, how it is distributed and the extent of health risks they may pose to human consumption specific to Enugu State, South-Eastern part of Nigeria. All the steps followed to carry out the entire analytical procedures in this study were consistent with published references. The end target was to understand better the status of goat carcass contamination with respect to the THMs, and if possible provide some insights about its toxicological safety.

### 2.2. Study Location and Sample Collection

The study location was Enugu State (6°51′24″ N and 7°23′45″ E), situated in the Southeast geo-political zone of Nigeria with an increasing population, realistically at six million [44]. Out of the slaughterhouses within the state, two major ones were purposively selected, namely the Nsukka and Kwata slaughterhouses. These two were selected given their capacity of processing more than 70% of goat carcass consumed in Enugu State [45].

The goat slaughter sections of the selected slaughterhouses were visited every other week for four months (December to January—dry season, and July to August—rainy/wet season). During each visit, about 5 goat carcasses, selected by a systematic random sampling method (one in five) were sampled. The first carcass selection was done by simple random sampling (toss of a coin). Thereafter, every fifth goat slaughtered was sampled. For each selected carcass, the sex was noted and the age estimated by the dentition method as described by Pace and Wakeman [46]. Thereafter, about 25 g each for kidney, liver and thigh muscle tissues were collected, separately packaged, labelled and transported to the laboratory of the National Centre for Energy Research and Development, University of Nigeria, Nsukka for the detection and quantification of As, Cd and Pb. The tissues′ collection and packaging was done with chemically cleaned instruments and the samples stored at freezing conditions (−20 °C) before the analysis. A total of 450 meat samples (150 each for kidney, liver and muscle tissues) from 150 randomly selected goat carcasses were analyzed.

### 2.3. Sample Preparation for Digestion

The frozen meat samples were allowed to thaw at room temperature (25–30 °C). Fat tissue components of the meat were manually removed and about 10 g oven-dried (Carbolite Gero, Pennsylvania, PA, USA) to constant weight at ~70 °C. The dried sample was granulated with mortar and piston (Changsha Jinto Co., Hunan, China), and was now ready for digestion and toxic metal determination.

### 2.4. Digestion and Toxic Metal Determinations

Wet digestion of the meat samples and subsequent analysis (Flame Atomic Absorption Spectrophotometry) for the presence of the THMs was performed as described by Nwude et al. [47]. Briefly, for each sample, ~3 g was weighed into a 100 mL digestion flask (Global spec., New York, NY, USA), then 30 mL of aqua regia [mixture of concentrated HNO_3_ (Sigma–Aldrich Corp., Missouri, MO, USA) and HCl (Sigma–Aldrich Corp., Missouri, MO, USA) in the ratio of 1:3 was added to digest the sample in a fume-cupboard at ~80 °C until a clear solution was obtained. The solution was then cooled, filtered into a standard volumetric flask using size 42 Whatman′s filter paper and made up to the 100 mL mark with deionized water. Thereafter, each solution was analyzed by flame atomic absorption spectrophotometry (AA-7000 Shimadzu, Kyoto, Japan; ROM 1.01) for the presence of As, Cd and Pb at their respective wavelengths.

### 2.5. Identification of Operational Parameters

The meat samples were quantitatively analysed for the THMs using the calibration curve method. Multi-element continuous calibration verification (CCV) standard 1, 100 mL (4400-010100–) (CPI International, California, CA, USA) was used to check the continued validity of the initial calibration. For each metal analysis, a blank sample solution was used to calibrate the instrument before testing the samples. Standard solution for each of the metal was prepared and fed into the spectrophotometer for automatic plotting of each analyses respective calibration curves. The elemental analysis in each of the samples was carried out in duplicates, and the average value computed and recorded. 

The limits of detection (LODs) were calculated as the concentration corresponding to three times the standard deviation (SD) of blanks, whereas the limits of quantification (LOQs) were computed as 10 times the SD of reagent blanks. Certified analytical grades of As, Cd and Pb (from Sigma-Aldrich^®^, Darmstadt, Germany) were analyzed, and the result compared with the label concentrations. 

All other chemicals and reagents used in this study were of analytical grade. Glassware were thoroughly washed with detergent and copiously rinsed with deionized water before use. Prior to the analysis, the qualitative and quantitative accuracy of the spectrophotometer was determined via metal recovery test (MRT). Certified reference materials, DORM-4 fish protein (National Research Council, Ottawa, ON, Canada), was used for the recovery.

The MRT also involved spiking analysed samples with a known quantity of each of the THMs. The spiked samples were subjected to chemical digestion as earlier described before the resultant solution was analysed for recovery and quantification of the metals. The operational parameters used for quantification of the metals, As, Cd and Pb, are as presented in Table 1.

### 2.6. Health Risk Assessment

The health risk assessment involved the measurements of estimated daily intake, target hazard quotient, hazard index, and target cancer risk. These were carried out with a view to estimate the various health effects associated with consumption of THMs in the meats. These are described below:

#### 2.6.1. Estimated Daily Intake (EDI)

The EDI of As, Cd and Pb through consumption of goat carcass in the study area were determined using the formula: EDI = [C × QMC]/BW as described by the United States Environmental Protection Agency (USEPA) [48]; where C = mean concentration of the specific metal in the meat (mg/kg) and BW = average body weight (kg). The QMC is the estimated quantity of goat carcass consumed daily (g) = 36 g as determined by survey of 104 randomly selected (goat carcass) consumers at slaughterhouses and meat markets in the study area. The average BW of children (0–17 years) and adults (≥18 years) were assumed to be 30 kg and 60 kg as set by USEPA [48]. The computed EDI values were then compared with the respective provisional tolerable daily intake (PTDI), of the respective THMs, which are amounts of the metals that can be ingested daily over a lifetime without appreciable health risk, as set by the joint *Food and Agriculture Organization of the United Nations and World Health Organization* (FAO/WHO) expert committee [49].

#### 2.6.2. Target Hazard Quotient (THQ)

The THQ, which is the non-carcinogenic health risks posed by consumption of the respective THMs was estimated as recommended by the US environmental protection agency [48]; using the formula: THQ = [(Ef × Ed × QMC × C) / (RfD × BW × Et)] × 10^−3^. The Ef = exposure frequency in days (365), Ed = exposure duration in a life time (70 years), QMC = quantity of goat carcass consumed (g), and C = mean concentration of the heavy metal (mg/kg). The RfD = oral reference dose of the metals was set by the USEPA [48] at 4 × 10^−3^ mg/kg/day for Pb, 1.0 × 10^−3^ mg/kg/day for Cd and 3 × 10^−3^ mg/kg/day for As. The BW= body weight (60 kg for an average adult) while Et = lifetime exposure (365 day × 70 years). The 70 years assumed for Ed and Et as well as the 365 days adopted for Ef were as recommended by the agency. If the calculated THQ value is <1, there is very little or no non-carcinogenic health risk. However, if the value is ≥1, then there is a possibility that non-carcinogenic adverse health effects may ensue following the (goat) meat consumption.

#### 2.6.3. Hazard Index (HI)

The facts that one food (meat) may be contaminated with multiple THMs and the consumption of the meat may result in simultaneous exposure to the metals make determination of HI imperative. The HI which is the summation of the individual THQs of the THMs detected in the meat was calculated as follows: HI = THQAs + THQCd + THQPb. When HI value is ≥1, it foretells that there is possibility of non-carcinogenic adverse health effects while values that are <1 depict very little or no non-carcinogenic effect. 

#### 2.6.4. Target Cancer Risk (TCR)

The TCR, which assesses the potential carcinogenic risks associated with lifetime exposure to carcinogens, was calculated as described by Naseri et al. [50] using the formula: TCR = CSF × EDI. The CSF is the cancer slope factor set at 0.008 mg/kg/day for Pb, 0.38 mg/kg/day for Cd and 1.5 mg/kg/day for As by the US Environmental Protection Agency [48].

### 2.7. Statistical Analysis

One-way analysis of variance (ANOVA) was applied to establish the differences in concentrations of metals in the tissues. Chi-square or Fisher’s exact test (as may be automatically highlighted by the software) was applied to determine if any significant associations existed between the occurrence of THMs and meat organs, age, sex and season. The emergent data have been presented in terms of mean values ± standard error of mean (SEM). The level of statistical significance was set at *p* < 0.05 (95% confidence interval). GraphPad Prism^®^ software, version 8.4.3 (GraphPad^®^ Inc., San Diego, California, CA, USA) was used to do the data analysis.

## 3. Results

### 3.1. Detection of Toxic Heavy Metals in Goat Carcasses

The percentage (%) detection of toxic heavy metals (THMs) in goat carcasses (*n* = 150) processed for human consumption in Enugu State, Nigeria, is shown in Table 2. At least one of the three THMs was detected in 56% (84/150) of the carcasses.

The statistical prevalence of the three THMs in goat carcasses (*n* = 450) processed for human consumption in Enugu State, Nigeria, is shown in Table 3. Cadmium, As and Pb were detected in 58.7% (264/450), 56% (252/450), and 32% (144/450) of the meat cuts, respectively. Apparent in most of the organs (kidney, liver and muscle), all three THMs, As, Cd and Pb were statistically different (*p* = 0.043).

### 3.2. Distribution of THMs in Goat Carcasses from Organs to Epidemiological Variables

The organs (*n* = 150) distributions of THMs above the maximum permissible limits in goats carcasses processed for human consumption in Enugu State, Nigeria, is shown in Table 4. Arsenic was detected in amounts above the WHO permissible levels in 24% (36/150) of the kidney, 28% (42/150) of the liver and 25.3% (38/150) of the muscle tissues. However, excessive amounts of Cd were recorded in 2.7% (4/150), 1.3% (2/150) and 0.7% (1/150) of the kidney, liver and muscle tissues, respectively. Overall, preponderance of As in the meats appeared more compared to Cd or Pb. Significant organ distributions of As (*p* = 0.041, χ^2^-value = 6.4) and Cd (*p* = 0.000, χ^2^-value = 15) were apparent, above the MPL.

The mean concentration of THMs specific to organs of goat carcasses processed for human consumption in Enugu State, Nigeria, is shown in Table 5. The metal concentrations ranged between 0.00 and 1.55 mg/kg for Pb, 0.00 and 1.807 mg/kg for As, as well as 0.02 and 0.829 mg/kg for Cd. The mean concentrations of As were 0.533 ± 0.103, 0.569 ± 0.091 and 0.452 ± 0.081 mg/kg for kidney, liver and muscle tissues, respectively. However, that of Pb was 0.480 ± 0.377, 0.450 ± 0.238 and 0.820 ± 0.388 mg/kg respectively for kidney, liver and muscle tissues. Moreover, no significant differences (*p* > 0.05) in THMs appeared across the organs studied.

The distribution of THMs found in goat carcasses (*n* = 150) according to various epidemiological variables is shown in Table 6. All the three toxic metals were prevalent in older (≥4 years) goat carcasses than in those aged less than four years. The age of the carcasses were statistically different (*p* < 0.05) across all three THMs detected (Table 6). The frequency of Cd detection appeared more in does/nannies (females) compared with the bucks (males). No statistical significance (*p* > 0.05) existed between the sex of the goats and the presence of Cd in the carcasses tested. With respect to seasonal distribution, the frequency of As and Pb detected during the rainy (wet) season differed significantly (*p* < 0.05) compared with those detected during the dry season. Other details on the epidemiological distributions of the metals are shown in Table 6 below.

### 3.3. Health Risk Assessment of Toxic Heavy Metals/Metalloids (THMs) in Goat Carcasses

The estimated daily intake (EDI) and target cancer risk (TCR) of THMs specific to children and adult consumers of goat carcasses processed in Enugu State, Nigeria, is shown in Table 7. The computed EDI for As in both the children and adult populations ranged between 0.271 and 0.640, which exceeded the WHO′s recommended PTDI. Similarly, the EDI values of Pb in both adult and children populations ranged between 0.270 and 0.984, which were above the WHO′s recommended PTDI. A similar trend was obtained for Cd, except that the 0.009 EDI value, computed for muscle tissue in the adult population, is less than the recommended PTDI. The calculated EDI values were generally higher for children than for the adult population. The TCR value obtained for Pb in muscle tissue was 1.4761. The target hazard quotient (THQ) and hazard index (HI) specific to children and adult consumers of goat carcass processed in Enugu State, Nigeria, is shown in Table 8. The THQ of all the THMs and in all the organs ranged between 0.009 and 0.246. The HI value for each of the three metals was less than unity.

## 4. Discussion

The THMs were detected at a high rate in goat carcasses slaughtered for human consumption in this study (refer to Table 2 and Table 3). This, in our opinion, might have a lot to do with the pollution status of the environment in which these goats were raised. The debate regarding the meats′ toxicological safety therefore continues given the adverse health effects that the THMs pose. Notably, at least one of the three THMs has been detected in the goat carcasses at this current study (refer to Table 3). This, in our opinion, might be attributable to indiscriminate disposal of industrial effluents, or excessive agricultural use of agrochemicals that accumulated THMs in pastures, fodder plants, drinking water sources, which eventually entered the animal’s tissues [18,32]. The contamination of grazing fields and water bodies with toxic metals can emanate from excessive use of organic fertilizers, the latter used to boost food production, and such agrochemicals may constitute heavy metals [51]. THMs bioaccumulating in plants enhances the possibility of (heavy metal) poisoning the herbivorous animals (that graze the plants), particularly goats. In mind that bio-magnification of THMs increases with trophic level [27], human that consume the contaminated meat are at greater risk of heavy metal poisoning and would suffer its deleterious health effects. Indeed, the implications of THMs bio-accumulation in edible plant and animal tissues are detrimental to food safety and endanger public health [17,32,52].

The frequent or inappropriate use of acaricides is among anthropogenic pathways that accounts for the high rate of the metals in goat meats. Such chemicals used to treat ectoparasite in FPAs may lead to tissue uptake of THMs, particularly As usually constituent of most ecto-parasiticide [3,53]. Extensive and semi-intensive types of goat husbandry methods, widely practiced in most developing countries, greatly expose the small ruminants to ectoparasitism and hence, frequent acaricidal treatments [54]. This might probably explain the preponderance of As in the sampled goat carcass compared to Cd or Pb (refer to Table 4 and Table 5) since most acaricides contain As [3,53]. Given their voracious and low-grazing feeding habits, goats remain naturally predisposed to heavy metal/metalloid intoxication, given the possibilities of either accidental geophagia, ingestion during grazing on a contaminated pastureland, and or drinking from THM-contaminated water body.

The prolonged bioaccumulation of THMs with ageing (of the animal) particularly in older goats [17] might likely be responsible for the increased distribution of As and Cd in the chevon of this current study (refer to Table 6). Especially at birth of FPAs, little to zero quantity of THMs may be detected. However, THMs has been shown with potential to increasingly accumulate with ageing (in both animals and humans) [3]. THMs accumulating in the tissues of FPAs could associate with the excretion rate falling below the acquisition rate. The THMs would bind with biomolecules and cellular components to achieve stable bonds/complexes, which may not be easily excreted [1,20,21]. Besides, seasonal predisposition of Cd in the goat carcass (refer to Table 5) might be due to latter′s predilection for green leafy vegetation (lush pasture) usually grazed during the rainy season [55,56]. The age-dependency and seasonality of THMs (refer to Table 6) offer useful epidemiological information, which has to be utilised so as to limit (THMs) contamination in goat meat. Chevon from young goats (less than four years) for human consumption could be a remedy, given that THMs increasingly accumulate with age [11,12,13,36]. Further, feeding goats concentrates or hay, rather than lush pasture, could help decrease THMs meat contamination in chevon. This is a strong proposition because THMs usually occupy the topsoil and lush pasture/fodder in contaminated pasturelands, particularly during the rainy season [56,57]. As goat production intensifies and cases of ectoparasitism rise, the frequency of acaricidal treatment will inevitably increase. Thus, to diminish the chances of meat contamination with THMs requires prudent use of heavy-metal-free acaricides to combat ectoparasites.

The fact that THMs were above the tolerance limits in goats slaughtered for human consumption (refer to Table 4) exemplifies the significant food safety and public health risks these metals do pose to the human population. For example, the exposures to excessive levels of Pb has been linked to such health concerns like distortions in hemoglobin synthesis, abnormalities in erythropoiesis and derangements in CNS functions [20,58], attention deficit/hyperactivity disorder, low intelligent quotient and dysfunctional behaviour in children and young adults [59,60]. In adults, there could be hypertension, cardiovascular diseases, mental retardation, behavioural disorders, neuropsychological deficits, early cognitive decline and other neurological disorders in cases of lead-poisoning [61,62,63]. 

We are concerned about the higher percentage of As dominating the THMs in the goat carcass at this current study given the potential adverse health effects it may pose to humans (refer to Table 2, Table 4, Table 5 and Table 6). It is equally this kind of food safety concerns associated with the accumulation of THMs in edible animal products that have made the international food safety regulatory authorities especially those under the European Commission (EC) [29] to set maximum permissible limits (MPL), or safety limits/tolerance levels, particularly for the metals in foods of animal origin. In line with this, if the level of THMs found in foods, especially edible animal proteins, are above the stipulated MPL, it would be deemed ‘unacceptable’ and ‘illegal’ given the adverse/substantial health effects/risks its exposures pose to consumer safety. If the human were to be exposed to THMs via the food chain, there are substantial health risks such toxic metals would pose, which would bring about adverse health effects [1,30,31,32]. 

Moreover, the As metalloid is well known to pose high health risks and remains highly carcinogenic. In fact, it is a very notable carcinogen understood to cause cancer following gastrointestinal or respiratory exposure [64,65,66]. Exposure to As was believed to have led to cancers of the skin, lungs, and bladder, according to the International Agency for Research on Cancer [67]. Although the exact mechanism of As-induced carcinogenesis appears not yet fully understood, there are a number of existing hypotheses that have been proposed that underscores the pathophysiology of its (As) toxicity. Examples include the induction of chromosome abnormalities, oxidative stress, altered DNA repair and altered DNA methylation patterns [65,67]. Monomethylarsonic acid, which is a major product of biomethylation of arsenic, is crucial in the arsenic-induced carcinogenesis [1,66,68].

The adverse human health effects of Cd metal, on the other hand, should not be underestimated. For instance, Cd exposure has been shown to bring about both taste and smell dysfunctions in China [69]. Excessive Cd level in the body can cause hepatotoxicity, osteoporosis, teratogenicity, neurotoxicity and immunosuppression due to apoptosis induction in normal human mononuclear cells [70,71]. The Cd metal has been implicated in the pathogenesis and pathophysiology of type-2 diabetes mellitus [8,72]. Induction of oxidative stress seems to be the major mechanism for the onset of deleterious health effects associated with Cd. Oxidative stress refers to tissue accumulation of reactive oxygen species (ROS) due to the decrease in the body’s ability to detoxify these reactive products following an assault. The ROS, to accrue from oxidative stress, could facilitate type-2 diabetes mellitus by decreasing pancreatic insulin production and increasing insulin resistance [5,71,73,74]. 

The mean concentrations of THMs in some of goat carcass samples, being above the safe limits, could result in adverse health effects. In particular, the amounts of As and Cd of this current study fell below 2.122 mg/kg (Cd) and (34.54 mg/kg (As) reported by Kulawik et al. [75] in Poland. However, the mean amounts of the metals detected were higher that the range concentrations of 0.001–0.104 mg/kg for As, 0.015–0.420 mg/kg for Cd and 0.003–0.100 mg/kg for Pb reported by Antoine et al. [76] in Jamaica. In Nigeria, the mean amounts of the THMs found were higher than the range concentrations of 0.014–1.37 mg/kg (Pb) and 0.00–0.00017 mg/kg (Cd) detected in street-vended foods in Benin City and Umunede [77]. On the contrary, our data fell below 28.28 mg/kg (Pb) and 0.38 mg/kg (Cd) found in chicken in Zamfara State [32]. The differences between data of current study and above mentioned reports could be due to variations in the levels of environmental contamination with the THMs. Despite these differences, the hazards posed by the THMs in foods remain vital given the public health risks even in minute quantities.

The health risk assessment of this current work has substantiated a very important information regarding Pb linked to children populations. That is, the TCR value of 1.476 computed for Pb in muscle tissues (refer to Table 7) might pose risk of cancer among children who consume goat carcass muscles from Enugu State. This is very relevant given that Pb toxicity and lead-poisoning cases in Nigerian children have been severally reported [78,79,80,81,82,83]. However, unlike the adult population, children in Enugu State may likely not be consuming chevon particularly on a daily basis. Hence, there is a high chance that any possible health hazard specific to children populations may not arise. Importantly, the HI and THQ values recorded less than one, thus, the consumption of chevon in the study area carries very little or no non-carcinogenic risk. Although most EDI values were above the PTDI set by the WHO for the respective THMs (refer to Table 7 and Table 8), adverse public health problems following the consumption of goat carcass in Enugu State may not be much because, according to Ladele et al. [84] beef meat has for long been more consumed over goat carcass. Despite this, screening tests against THMs in Nigerian slaughterhouses should be instituted so as to ascertain the toxicological safety of meats intended for human consumption.

## 5. Conclusions

The detection, distribution, and health risk assessment of THMs, specifically As, Cd, and Pb in goat carcasses processed for human consumption in South-Eastern Nigeria, with reference to Enugu State, were successfully investigated. For emphasis, the current status of goat carcass contamination with THMs, and insights into its toxicological safety, as it pertains to the meat consumers, have been successfully discussed. We have demonstrated that excessive levels of As, Cd and Pb can be detected in edible tissues of goat carcasses processed for human consumption, specific to the South-Eastern part of Nigeria. The presence of excessive levels of As, Cd and Pb poses significant health risks to the meat consumers considering the adverse health effects and bio-accumulative potentials of THMs. We also showed that the EDI for all the metals/metalloids were above the PDTI set by the WHO of the United Nations. Despite the high EDI values, the possible consequential health problems may not be too much since chevon appears not consumed daily. The THQ and HI for all the three THMs in all the organs fell below unity, which was indicative of little or no non-carcinogenic risks.

It is essential to reiterate herein that as THMs increasingly build up in the environment, either from contamination of drinking water sources and pastureland, or excessive use of acaricides used to treat ectoparasites involved in goat production, a number of health risk issues would continually arise. When THMs accumulate in this context, they will be detected in goat carcass being processed and sold for human consumption, and it is only through studies like this, herein, would the health risks it pose be conveyed. Indeed, the cumulated health effect/risks on those who consume the infected chevon, from oxidative stress, inhibition of enzymes, organ damage, cancer, poor sight/taste/smell, among others, can be significant. We have, at this current study, acquired an improved understanding about the current status of goat carcass contamination with THMs, and this is specific to the South-Eastern Nigeria. We have also provided additional insights about its toxicological safety. Indeed, the information assembled herein can contribute in building the policy framework and formulation regarding public health safety in Nigerian slaughterhouses.

Essentially: at the slaughterhouse level, the processed meats should be increasingly screened for chemical (residue) contamination, particularly THMs, to ensure compliance with internationally recognized safety limits. Given the adverse health problems associated with THMs’ contamination of goat carcass, there is need for the direction of future studies to evaluate how the indiscriminate use of agrochemicals and other anthropogenic activities impact on pastureland and drinking water sources used for livestock production. Another direction of future studies should be on assessing how farmers over time periods and at various locations have used agrochemicals on the pastureland/goat production, so as to decipher the prevailing factors that impact THMs on its excessive use with respect to livestock production, which would have health risk implications on emergent meat product and consumer safety. The evaluation of such factors would require robust statistical tools, regression models, and principal component analysis, to offer predictive location/time-related insights, which would be useful for the Nigeria federal/state government public health-THMs policy framework. Through this, the environmental contamination by THMs would be minimised, even in meat products.

## Figures and Tables

**Table 1:** Operational parameters used for quantification of arsenic, cadmium and lead in edible tissues of goats slaughtered for human consumption in Enugu State, Nigeria.

Parameter	Heavy Metals		
	Arsenic	Cadmium	Lead
Wavelength (nm)	193.7	228.8	283.3
Slit with (nm)	0.7	0.7	0.7
Limit of detection (mg kg^−1^)	0.0005	0.0001	0.0001
Limit of quantification (mg kg^−1^)	0.002	0.001	0.001
Metal recovery range (%)	98–101.4	98.2–101.5	97–100.9

**Table 2:** Percentage (%) detection of toxic heavy metals (THMs) in goat carcasses (*n* = 150) processed for human consumption in Enugu State, Nigeria.

Detection Status of Toxic Heavy Metals in Goat Carcasses	Number of Goat Carcasses	Percentage (%) of Goat Carcasses
Toxic heavy metals or metalloids detected	84	56
No Toxic heavy metals or metalloids detected	66	44
Total (*n*)	150	100

**Table 3:** Statistical prevalence of toxic heavy metals (THMs) in goat carcasses (*n* = 450) processed for human consumption in Enugu State, Nigeria.

THMs	Number Positive	Prevalence	χ^2^-Value	*p*-Value
Arsenic (As)	252	56	12.2	0.043 *
Cadmium (Cd)	264	58.7		
Lead (Pb)	144	32		

* Denotes statistical significance, Chi-square test; GraphPad Prism^®^ version 8.4.3.

**Table 4:** Organs (*n* = 150) distributions of toxic heavy metals (THMs) above the maximum permissible limits (MPL) in goats carcasses processed for human consumption in Enugu State, Nigeria.

THMs	Organs	Number Contaminated Above MPL (%) **	χ^2^-Value	*p*-Value
Arsenic (As)	Kidney	36 (24)	6.4	0.041 *
	Liver	42 (28)		
	Muscle	38 (25.3)		
Cadmium (Cd)	Kidney	4 (2.7)	15	0.000 *
	Liver	2 (1.3)		
	Muscle	1 (0.7)		
Lead (Pb)	Kidney	22 (14.7)	0.65	0.724
	Liver	22 (14.7)		
	Muscle	24 (16)		

* Denotes statistical significance, Fisher′s exact test; GraphPad Prism^®^ version 8.4.3; ** value outside bracket refers to the actual number of samples; value inside bracket refers to percentage value from total number.

**Table 5:** Mean concentration of toxic heavy metals (THMs) specific to organs of goat carcasses processed for human consumption in Enugu State, Nigeria.

THMs	Range Conc. (mg/kg)	Mean Concentrations ± SEM (mg/kg) in Organs			*p*-Value
		Kidney	Liver	Muscle	
Arsenic (As)	0.00–1.81	0.53 ± 0.10	0.57 ± 0.09	0.45 ± 0.08	0.651
Cadmium (Cd)	0.020–0.83	0.06 ± 0.32	0.02 ± 0.00	0.02 ± 0.00	0.270
Lead (Pb)	0.00–1.55	0.48 ± 0.38	0.45 ± 0.24	0.82 ± 0.39	0.697

Maximum permissible limits: As = 0.1 mg/kg; Cd = 0.05 mg/kg; Pb = 0.1 mg/kg.

**Table 6:** Distribution of toxic heavy metals/metalloids (THMs) according to various epidemiological variables in goat carcasses (*n* = 150) processed for human consumption in Enugu State.

THMs	Epidemiological Variables	Number Sampled	Number Positive	Prevalence	Odds Ratio	95% CI	*p*-Value
Arsenic	*Age*						
	<4 years	70	31	44.3	0.39	0.20–0.76	0.005 *
	≥4 years	80	53	66.3			
	*Sex*						
	Male	66	38	57.6	1.1	0.56–2.10	0.730
	Female	84	46	54.8			
	*Season*						
	Wet/rainy	63	39	61.9	1.5	0.78–2.9	0.215
	Hot/dry	87	45	51.7			
Cadmium	*Age*						
	<4 years	70	34	48.6	0.32	0.16–0.63	0.001 *
	≥4 years	80	50	62.5			
	*Sex*						
	Male	66	32	48.5	0.58	0.30–1.11	0.103
	Female	84	52	61.9			
	*Season*						
	Wet/rainy	63	44	69.8	2.4	1.1–4.5	0.018 *
	Hot/dry	87	40	45.0			
Lead	*Age*						
	<4 years	70	47	67.1	2.4	1.2–4.6	0.010 *
	≥4 years	80	37	46.3			
	*Sex*						
	Male	66	34	51.5	0.72	0.4–1.4	0.327
	Female	84	50	59.2			
	*Season*						
	Wet/rainy	63	49	77.8	5.2	2.5–10.8	0.000 *
	Hot/dry	87	35	40.2			

* Denotes statistical significance, based on Chi-square test, CI = Confidence Interval.

**Table 7:** Estimated daily intake (EDI) and target cancer risk (TCR) of toxic heavy metals (THMs) specific to children and adult consumers of goat carcass processed in Enugu State, Nigeria.

THMs	Organs	EDI		TCR	
		Children	Adult	Children	Adult
Arsenic	Kidney	0.640 *	0.320 *	0.0051	0.0026
	Liver	0.683 *	0.341 *	0.0055	0.0027
	Muscle	0.542 *	0.271 *	0.0043	0.0022
Cadmium	Kidney	0.066 *	0.033 *	0.0251	0.0125
	Liver	0.022 *	0.011 *	0.0084	0.0042
	Muscle	0.019 *	0.009	0.0072	0.0036
Lead	Kidney	0.576 *	0.288 *	0.8640	0.4320
	Liver	0.540 *	0.270 *	0.8100	0.4051
	Muscle	0.984 *	0.492 *	1.4761 **	0.738

* EDI values that exceeded the WHO′s provisional tolerable daily intake (PTDI). The PTDI for As = 0.003 mg/kg/day, Cd = 0.001 mg/kg/day and Pb = 0.002 mg/kg/day. ** TCR value > 1 = carcinogenic risk.

**Table 8:** Target hazard quotient (THQ) and hazard index (HI) specific to children and adult consumers of goat carcass processed in Enugu State, Nigeria.

Organs	THQ						HI	
	As		Cd		Pb			
	Children	Adults	Children	Adults	Children	Adults	Children	Adults
Kidney	0.213	0.107	0.066	0.032	0.144	0.072	0.423	0.211
Liver	0.228	0.114	0.022	0.011	0.135	0.068	0.385	0.193
Muscle	0.182	0.091	0.019	0.009	0.246	0.123	0.447	0.223

THQ or HI values less than one indicates no or very little non-carcinogenic health risk.

## Data Availability

Data sharing not applicable.
